# Supplementary material for: Reconstruction and Removal Mechanisms of Gel-like Membrane Fouling for Seawater Desalination: Experiments and Molecular Dynamics Simulations
Source: Polymers (Basel). 2022 Sep 7;14(18):3734. doi: 10.3390/polym14183734 (PMC9500861; doi:10.3390/polym14183734)
Supplement: Supplementary file 1 [file polymers-14-03734-s001.zip › polymers-1868847-supplementary.pdf]

polymers MDPI

Article

Reconstruction and Removal Mechanisms of Gel-like Membrane Fouling for Seawater Desalination: Experiments and Molecular Dynamics Simulations

Qi Wang [1,2], Xiang-Yu Yang [2], Rong-Hui Qi [2,*] and Li-Zhi Zhang [2]

[1] School of Electric Power Engineering, South China University of Technology, Guangzhou 510640, China
[2] Key Laboratory of Enhanced Heat Transfer and Energy Conservation of Education Ministry, School of Chemistry and Chemical Engineering, South China University of Technology, Guangzhou 510640, China
* Correspondence: qirh@scut.edu.cn; Tel.: +86-020-87112053

Supplementary Materials: The following supporting information can be downloaded at: https://www.mdpi.com/article/10.3390/polym14183734/s1.

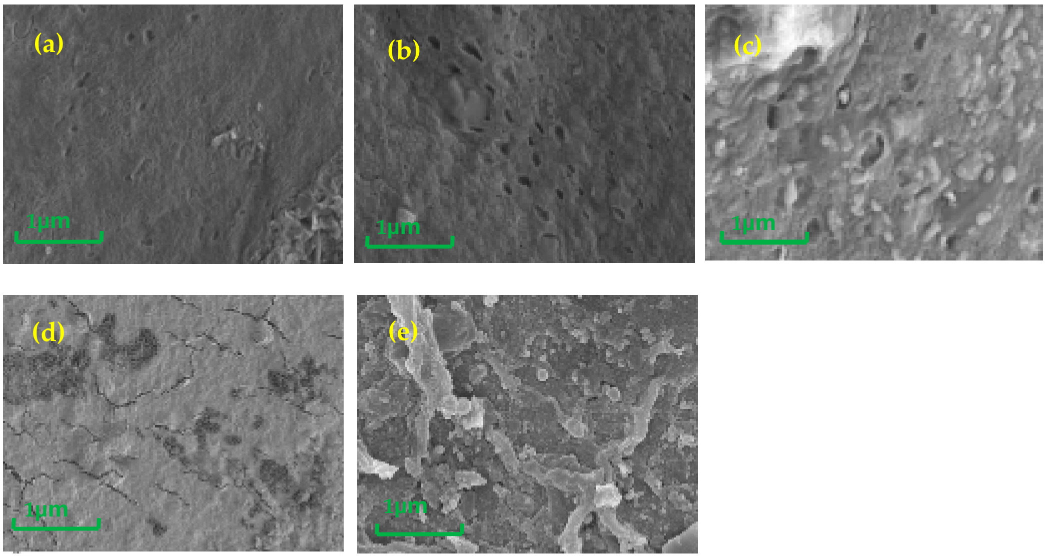

Figure S1. The pore size of gel layer.

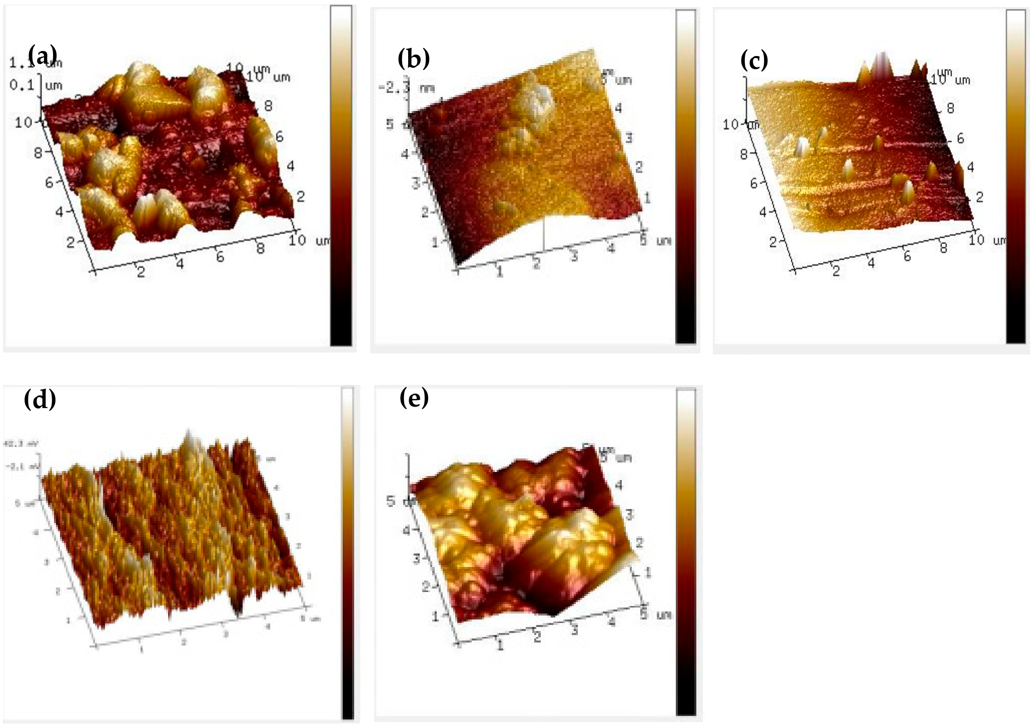

Figure S2. The AFM images of the pollutant gel layer: (**a**) The concentration of organic acid was 2%; (**b**) The concentration of organic acid was 2.5%; (c) The concentration of organic acid was 3%. (d) The concentration of organic acid was 3.5%; (e) The concentration of organic acid was 4%.

Table S1. The elastic stiffness matrix (GPa) of the gel.

24.2133	8.8583	9.9454	-0.3980	0.3127	0.2524
8.8583	25.7148	9.3042	0.0528	0.0662	0.6521
9.9454	9.3042	23.4086	-0.7025	-0.5777	0.2322
-0.3980	0.0528	-0.7025	5.8946	-0.0417	-0.0586
0.3127	0.0662	-0.5777	-0.0417	6.9433	0.0375
0.2524	0.6521	0.2322	-0.0586	0.0375	7.3565
